# Improved Real-Time Semantic Segmentation Network Model for Crop Vision Navigation Line Detection

**DOI:** 10.3389/fpls.2022.898131

**Published:** 2022-06-02

**Authors:** Maoyong Cao, Fangfang Tang, Peng Ji, Fengying Ma

**Affiliations:** School of Information and Automation Engineering, Qilu University of Technology (Shandong Academy of Sciences), Jinan, China

**Keywords:** precision agriculture application, visual navigation, semantic segmentation, crop rows detection, navigation path recognition

## Abstract

Field crops are generally planted in rows to improve planting efficiency and facilitate field management. Therefore, automatic detection of crop planting rows is of great significance for achieving autonomous navigation and precise spraying in intelligent agricultural machinery and is an important part of smart agricultural management. To study the visual navigation line extraction technology of unmanned aerial vehicles (UAVs) in farmland environments and realize real-time precise farmland UAV operations, we propose an improved ENet semantic segmentation network model to perform row segmentation of farmland images. Considering the lightweight and low complexity requirements of the network for crop row detection, the traditional network is compressed and replaced by convolution. Based on the residual network, we designed a network structure of the shunting process, in which low-dimensional boundary information in the feature extraction process is passed backward using the residual stream, allowing efficient extraction of low-dimensional information and significantly improving the accuracy of boundary locations and row-to-row segmentation of farmland crops. According to the characteristics of the segmented image, an improved random sampling consensus algorithm is proposed to extract the navigation line, define a new model-scoring index, find the best point set, and use the least-squares method to fit the navigation line. The experimental results showed that the proposed algorithm allows accurate and efficient extraction of farmland navigation lines, and it has the technical advantages of strong robustness and high applicability. The algorithm can provide technical support for the subsequent quasi-flight of agricultural UAVs in farmland operations.

## Introduction

With the rapid development of precision agriculture, agricultural modernization equipment is increasingly developing in the direction of intelligence (Romeo et al., 2012). The mechanization, automation, and informatization of agricultural equipment has reached a high level, and numerous notable achievements have been made. However, existing agricultural equipment still requires manual driving operation, and the labor intensity for the driver is still very high for large-scale operation tasks. With the continuous improvement of Internet of Things (IoT) technology, UAV technology has developed rapidly (Alsamhi et al., 2021a). The main applications of UAVs in the agricultural field include surveying and mapping, spraying, sowing, and monitoring of pests and diseases (Alsamhi et al., 2021b). Agricultural UAV technology has the characteristics of intelligence, high operation efficiency, and no terrain restrictions. By adopting drones, crop yields can be increased, time and effort can be saved, and the return on investment can be significantly maximized. Therefore, it is of great significance to fully consider the characteristics of UAVs in farmland operations to promote the development of precision agriculture.

Autonomous navigation technology is crucial for agricultural flying robot equipment. It can effectively reduce the labor intensity of agricultural machinery drivers, increase the profit and efficiency of field operations, and improve operational safety. The current advanced navigation technologies include sensor-based laser detection technology, satellite navigation, positioning technology, and visual navigation technology. Satellite navigation and positioning technology can be used to perform planning for a wide range of operating areas; however, it is limited by poor flexibility, susceptibility to signal interference, and no capacity to adapt to various regional changes (Grewal et al., 2007). Sensor-based laser detection technology mainly relies on laser, ultrasonic, or visual sensors to perceive the surrounding environment. However, these sensors can only measure distance information, cannot perceive color information, and cannot meet the operational requirements of complex farmland environments (Winterhalter et al., 2018). Visual navigation technology can be used to collect farmland images through cameras and process them to obtain navigation paths. By contrast, this technology has broad detection information, complete information acquisition, and price advantages and is becoming a research hotspot (Yasuda et al., 2020).

Crop row detection is a critical element for the development of vision-based navigation in agricultural flying robots. The application of machine vision algorithms to extract crop row baselines quickly and accurately is a critical area for improvement (Basso and Pignaton de Freitas, 2020). In the early days, Guerrero and Ma implemented inter-row segmentation based on color segmentation and clustering algorithms to extract navigation lines (Guerrero et al., 2013; Ma et al., 2021); however, the resulting effect was not ideal. Tu et al. (2014) proposed a method to detect crops using a quad. Through the movement, expansion, and contraction of quadrilaterals, they detected the surrounding crop ridges. This method considerably reduces the computation and time cost, but the accuracy is relatively low. Meng et al. (2018) researched agricultural mobile robots with monocular vision in the natural environment. They proposed an improved genetic algorithm that encodes two random points at the top and bottom of the crop row images as chromosomes to identify guidelines quickly and accurately. The algorithm showed good adaptability to different growth stages and different crops. However, these methods based on machine learning are easily affected by environmental factors such as light and weeds, resulting in poor robustness and inaccurate extraction of navigation lines. Simultaneously, flying robots adjust the path in real-time during the travel process. The amount of image data information is continuously superimposed, making the device’s processing slower, resulting in a low real-time performance that cannot meet the actual requirements.

In recent years, deep learning in the field of machine vision has developed rapidly, and the convolutional neural network (CNN) algorithm, which is a hotspot of current research, has achieved remarkable results (Dhaka et al., 2021). Kundu et al. (2021) proposed the fusion of IoT with deep transfer learning to analyze image and digital data. Wieczorek et al. (2021) proposed lightweight CNN construction, where the number of necessary image processing operations was minimized. CNN algorithms have overcome the main challenges of implementing vision-based navigation systems. They are widely employed in various agricultural vision tasks and have provided promising results (Hong et al., 2020; Saleem et al., 2021). The semantic segmentation network of deep learning is a powerful image segmentation algorithm that can be applied to the more complex farmland environment in the agricultural field and provides very good results (Lin and Chen, 2019).

Based on existing research, this paper proposes a new semantic segmentation model for crop line images. The novelty of our model lies in designing the network structure for shunt processing and performing compression and convolution replacement processing operations on the traditional ENet. By introducing the residual flow to record the boundary information in the image, the accuracy of crop row boundary location and crop row segmentation can be improved. After that, in the process of using RANSAC to fit the navigation line, a new model scoring index is defined according to the characteristics of the segmented image, which can accurately extract the visual navigation line in real-time.

The remainder of this article is organized as follows. Section Related Work discusses related work on agricultural drones and crop row detection. Section Methodology describes the methodology and theory proposed in this paper. Section Experiment presents the results of the study, and Section Conclusion concludes the paper.

## Related Work

UAVs have the characteristics of maneuverability and flexibility. They are relatively unaffected by weather when equipped with sensors for farmland monitoring, and high-resolution images can be obtained. They are of great significance in assisting the precise management of farmland and are conducive to improving farmland operation efficiency (Alsamhi et al., 2018; Gupta et al., 2020). In recent years, the application of UAVs to agricultural production management has become a research hotspot, and successive studies have been conducted. Combining drones with IoT, Almalki et al. (2021) proposed a low-cost integrated platform to help improve the crop productivity, cost-effectiveness, and timeliness of farm management. Nebiker et al. (2016) used low-altitude drones equipped with high-resolution cameras and multispectral sensors to accurately and quickly detect infection in potato fields. Faiçal et al. (2017) proposed a coordinated spraying system of drones and wireless sensor networks, which can appropriately determine and change the routes and actions of drones according to weather conditions and effectively manage the pesticide spraying process. Dai et al. (2017) proposed a drone spraying system specially designed for fruit on trees. The system includes a vision system for target recognition and a navigation system to control the drone.

Field crops are generally planted in rows, so accurate extraction of crop rows is the basis of UAV visual navigation. With the great research progress of deep learning in image processing, crop row detection based on semantic segmentation networks is the focus of modern scholars. In the field of deep learning-based crop row detection, Lin and Chen (2019) introduced fully convolutional networks (FCNs) and efficient neural network (ENet) for the semantic segmentation of tea planting scenes and obtained the outline and obstacles of the tea tree row, which greatly improved the real-time performance of the tea picking machine. Lan et al. (2020) proposed a novel global context-based dilated CNN to perform semantic segmentation and extract the road. Adhikari et al. (2020) proposed a new method for crop row detection in paddy fields, in which semantic graphs were introduced to annotate crop rows. The graphs were then input into a deep convolutional encoder-decoder network and used as a template matching algorithm to extract the position of rice rows. The tractor was controlled for precise autonomous navigation in rice fields. Bakken et al. (2021) proposed a CNN-based semantic segmentation network for crop row detection. The algorithm automatically uses noisy labels to train the network, enabling the robot to adapt well to seasonal and crop changes. The network segmentation accuracy enables the vision-based movement of agricultural robots along crop rows.

## Methodology

The visual guideline recognition method proposed here mainly has two key steps: interline semantic segmentation and guideline fitting. Inter-row semantic segmentation adopts the improved ENet structure, and pixel-level recognition is performed on farmland images to accurately extract the crop row’s position. Navigation line fitting is based on the semantic segmentation results, and the random sampling consensus algorithm is used to fit the crop row centerline and then draw the visual navigation line.

### Crop Row Segmentation

ENet is a real-time lightweight semantic segmentation network with low computational complexity and relatively low computational requirements (Paszke et al., 2016). This network is both accurate and fast, and its design is suitable for crop row segmentation.

**Figure 1:**
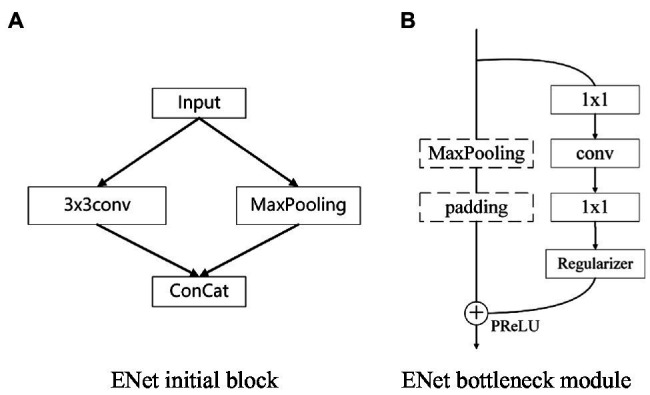
Module structure diagram.

The model architecture of the traditional ENet mainly includes an initialization stage and five bottleneck stages. The first three bottleneck stages are used for encoding, and the last two are used for decoding. The network includes an initialization module and a bottleneck module. The module structure is shown in Figure 1. The initialization module can compress the input and reduce visual redundancy. The bottleneck module uses the idea of residual connection and is divided into a main branch and an extended branch. When downsampling, the main branch will add a maximum pooling layer and a padding layer, reducing the total number of parameters and the number of calculations, which can improve the overall speed. It uses a relatively symmetric encoder-decoder structure and traditional downsampling and upsampling for training. This structure cannot effectively transfer semantic information for subsequent upsampling, resulting in low model accuracy.

**Figure 2:**
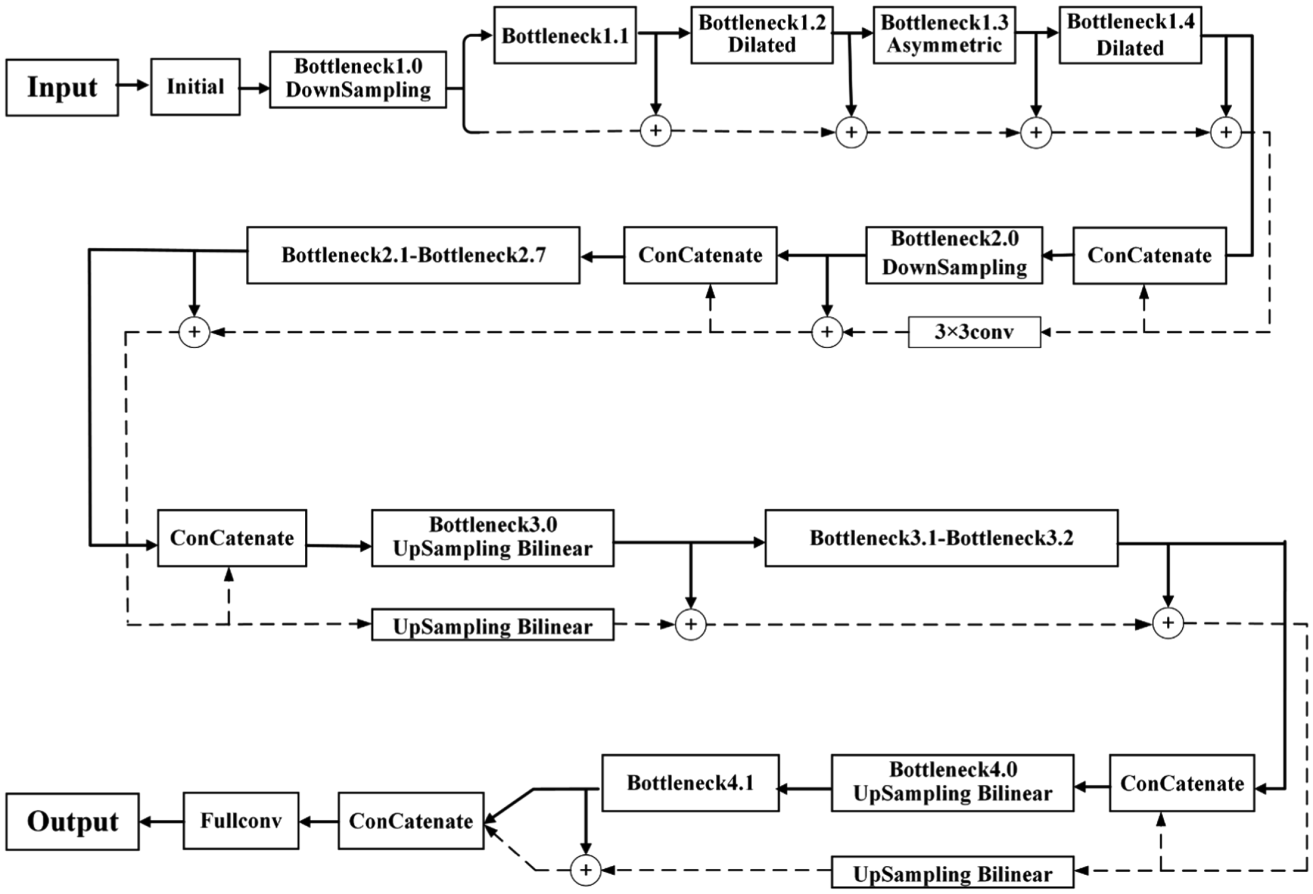
The improved ENet structure.

Based on the above problems, we designed a new network structure, adding two processing streams—a pooling stream and a residual stream—to the encoding and decoding stages. The pooling stream is used to obtain high-dimensional semantic information, and the residual stream is used to record low-dimensional boundary information to ensure that it is more suitable for farmland image segmentation. To further improve the running speed of the network and reduce the complexity of the model, we performed model compression and convolution replacement. The improved and optimized network structure is shown in Figure 2.

#### Network Structure of Offload Processing

ENet only focuses on extracting high-dimensional semantic information, ignoring the low-dimensional boundary information contained in feature maps after the initial convolution, which results in low segmentation accuracy. The low-dimensional boundary information includes numerous image details, which can help the network perform accurate segmentation. Therefore, a network structure of the shunting process is designed, and the residual stream is used to accumulate the low-dimensional boundary information of the image, which makes the features more noticeable and facilitates the application in the later decoding stages.

After Bottleneck1.0 downsampling, the initial pooling feature map is obtained. Based on this feature map, more low-dimensional boundary details can still be retained. Therefore, it is used as the initial residual feature map of the residual stream branch. In Bottleneck1.1–1.4, the branching process of the pooling stream is the same as that of the traditional ENet. The residual stream branch accumulates the result of each pooling stream operation with the previous residual feature map to obtain a new residual feature map, to ensure that the boundary information of each stage can be recorded. Before entering the new bottleneck stage, the pooling feature map output from the previous stage and residual feature map are channel-fused as the input of the pooling stream branch. Bottleneck2.0 performs downsampling. Thus, the size of the output pooling stream feature map changes. Therefore, before feature fusion, a 3×3 convolution operation is added to the residual branch to expand the residual feature map to the same size.

To solve the loss of boundary information caused by image downsampling in the decoding stage, ENet uses the maximum index generated by downsampling twice in the encoding stage to restore the boundary information if the picture is insufficient. The proposed network uses a residual stream to record boundary information; thus, the maximum pooling upsampling is discarded. Instead, the main branch in the bottleneck module is replaced by a bilinear interpolation operation for the upsampling operation. Simultaneously, the residual stream corresponding to this stage is also upsampled by bilinear interpolation; thus, the low-dimensional boundary information accumulated by the residual stream is not easily lost. After upsampling is completed, the final pooling stream feature map and the residual stream feature map are connected, and the result is input into the fully connected layer to obtain a feature classification map of the same size as the original input image. Finally, the feature map is classified into two categories to complete the entire segmentation process.

#### Convolution Replacement

**Figure 3:**
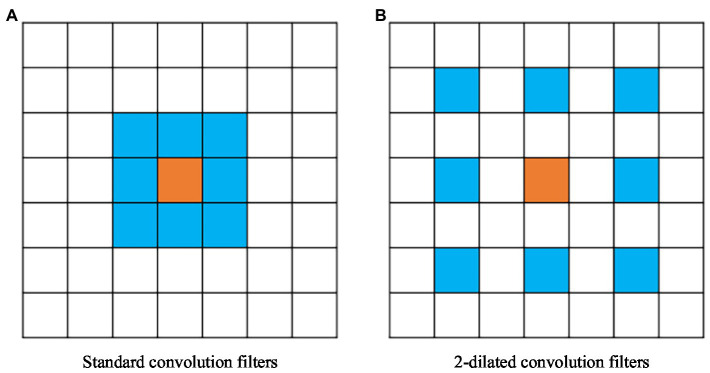
Dilated convolution. **(A)** A standard convolution kernel of 3×3, and the size of its corresponding receptive field is 3×3. **(B)** A 3×3 convolution kernel with a dilation rate of 2, and the size of the corresponding receptive field is 5×5.

The encoder in the traditional ENet contains two downsampling operations. The role of downsampling is to reduce the image resolution, reduce the redundancy of information, and speed up image processing. The convolution operation can be performed on the downsampling feature to obtain a larger receptive field and collect more contextual information. To improve the problem of a reduced receptive field caused by the small number of downsampling processes, we improve the four ordinary convolutions after the first downsampling, and introduce dilated convolution. Dilated convolution is performed by inserting “0” in the convolution kernel, which increases the receptive field without adding additional parameters, and can obtain more contextual information, thereby improving the segmentation accuracy of the network. Dilated convolution is shown in Figure 3, where Figure 3A is a standard convolution kernel of 3×3, and the size of its corresponding receptive field is 3×3. Figure 3B is a 3×3 convolution kernel with a dilation rate of 2, and the size of the corresponding receptive field is 5×5.

Referring to the arrangement of convolution modules in the second stage of the traditional ENet, we replace the convolution modules in the first stage of the pooling stream processing and replace the three standard convolution modules in bottleneck 1.2–1.4 with expansion. Dilated convolution with a rate of 2, asymmetric convolution with a size of 5, and dilated convolution with a dilation rate of 4 result in a new pooling feature map. Convolutions are replaced by an equal number, thus not affecting the speed of the algorithm.

#### Model Compression

The second and third stages in the traditional ENet are repeated convolution operations on the same scale, which may cause information redundancy problems. In general, repeated convolution operations repeatedly extract feature information, resulting in certain information redundancy (Brostow et al., 2008). Furthermore, downsampling is not performed in the third stage. That is, no effective semantic information is extracted. Thus, this study removes the third part of the repeated convolution operation of the ENet, further reduces the number of parameters, and improves network processing speed to meet real-time requirements.

### Navigation Line Fitting

#### Feature Point Extraction

The premise of obtaining the visual navigation line is to extract feature points of the crop row area. In this study, the segmented network’s binary image is horizontally scanned at equal intervals. The average value of the abscissa of the intersection of the white edge and the scanning line is used as the abscissa of the feature point, and the product of the scanning order and the interval is used as the ordinate. The calculation formula is given as follows:


(1) $$\left\{ \begin{array}{c} x=\frac{1}{k}\overset{k}{\underset{i=1}{\sum }}x_{i}\\ y=hN \end{array}\left(0\leq k\leq \frac{\left[H_{img}\right]}{h}\right)\right.$$

where *k* represents the number of intersections between the white edge and the scan line, $x_{i}$ represents the abscissa of the intersection of the white edge and the scan line, *h* represents the interval of the scan line, *N* represents the scan order, and $H_{img}$ represents the height of the image. Traversing each row of an image results in a large time and memory overhead. Therefore, the value of the scanning interval *h* should be reasonably selected. Experiments show that *h* is set to 8, which ensures real-time detection without affecting accuracy.

#### Navigation Line Fitting

**Figure 4:**
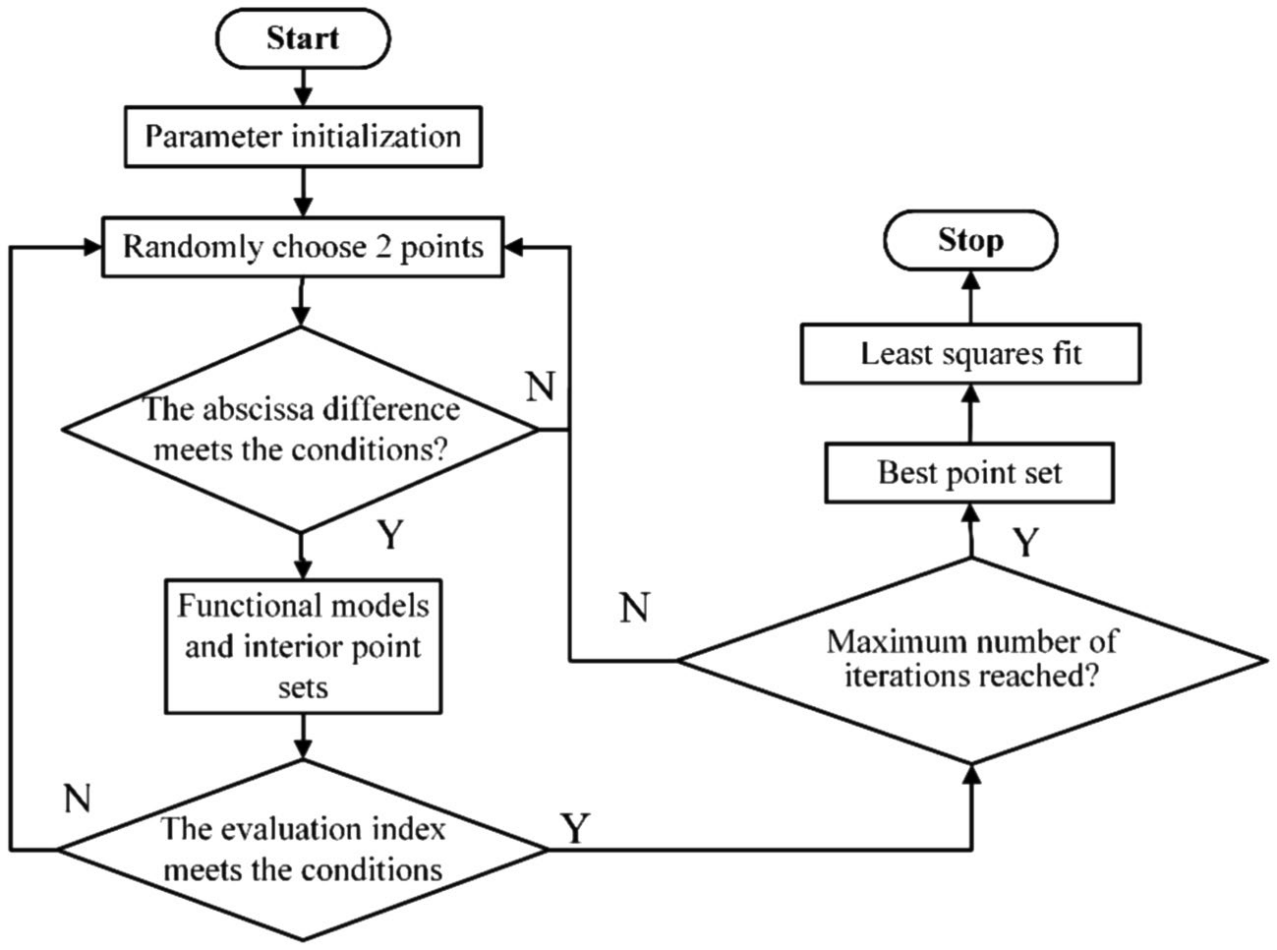
Improved RANSAC algorithm flow chart.

The existing navigation line fitting methods mostly use the Hough transform and the least-squares method for straight-line fitting. However, the effect is not ideal when there are many noise points, and problems of incomplete line detection and large deviation occur. We adopted the RANSAC algorithm, a robust model estimation method commonly used in curve fitting and widely used in image processing. Using the RANSAC algorithm, the edge information points are eliminated to obtain the optimal interior-point set, and then, the visual navigation line is fitted according to the least square method. Figure 4 presents a flowchart of the algorithm.

The traditional RANSAC algorithm uses a completely random method to select two points to build a model, which is time-consuming. Because the initial point is not constrained, it is possible to select two pixels that do not belong to the same crop row area; thus, the obtained linear model parameters are wrong, and the time consumed for subsequent evaluation of the model parameters becomes redundant. Therefore, in the proposed navigation line fitting algorithm, when randomly selecting two initial pixel points $P_{1}\left(x_{i},y_{i}\right)$ and $P_{2}\left(x_{j},y_{j}\right)\text{,}$ to limit the range of absolute difference between the abscissas of any two pixels, it should satisfy the following:


(2) $$|x_{i}-x_{j}|>T$$

where *T* is the set coordinate threshold. The distance threshold was set to one third of the image width in this study. If the coordinate threshold condition is satisfied, the mathematical model is calculated according to the two sample points, that is, the direction vector of the straight line between the two points. Then, the distance of all other points to that line is calculated. The points within the distance threshold are specified as inliers, and the number of inliers is counted. Considering that the real navigation line is generally a long straight line, a model scoring index is defined as follows:


(3) $$\mathrm{score}=m_{t}\left(l+p\right)$$

where *score* represents the model scoring index, $m_{t}$ represents the number of all inliers, *l* represents the length of the straight line generated by the model, and *p* is a default parameter. Experiments determined *p* to be 15. In the iterative process, if the scoring criteria meet the conditions, the optimal model has been found, and the iteration is stopped. All the interior points at this time constitute the optimal interior-point set. Then, the least-squares method is used to fit the centerline of each crop row to the optimal point set and finally extract the navigation line.

## Experiment

### Hardware Equipment

The computer used for the experiments had the following configuration: Windows 10 operating system, an Intel(R) Xeon Gold 5,118 CPU, a reference frequency of 2.30 GHz, 512 GB memory, and a GeForce RTX 2080Ti 11GB GPU.

### Dataset Preparation

**Figure 5:**
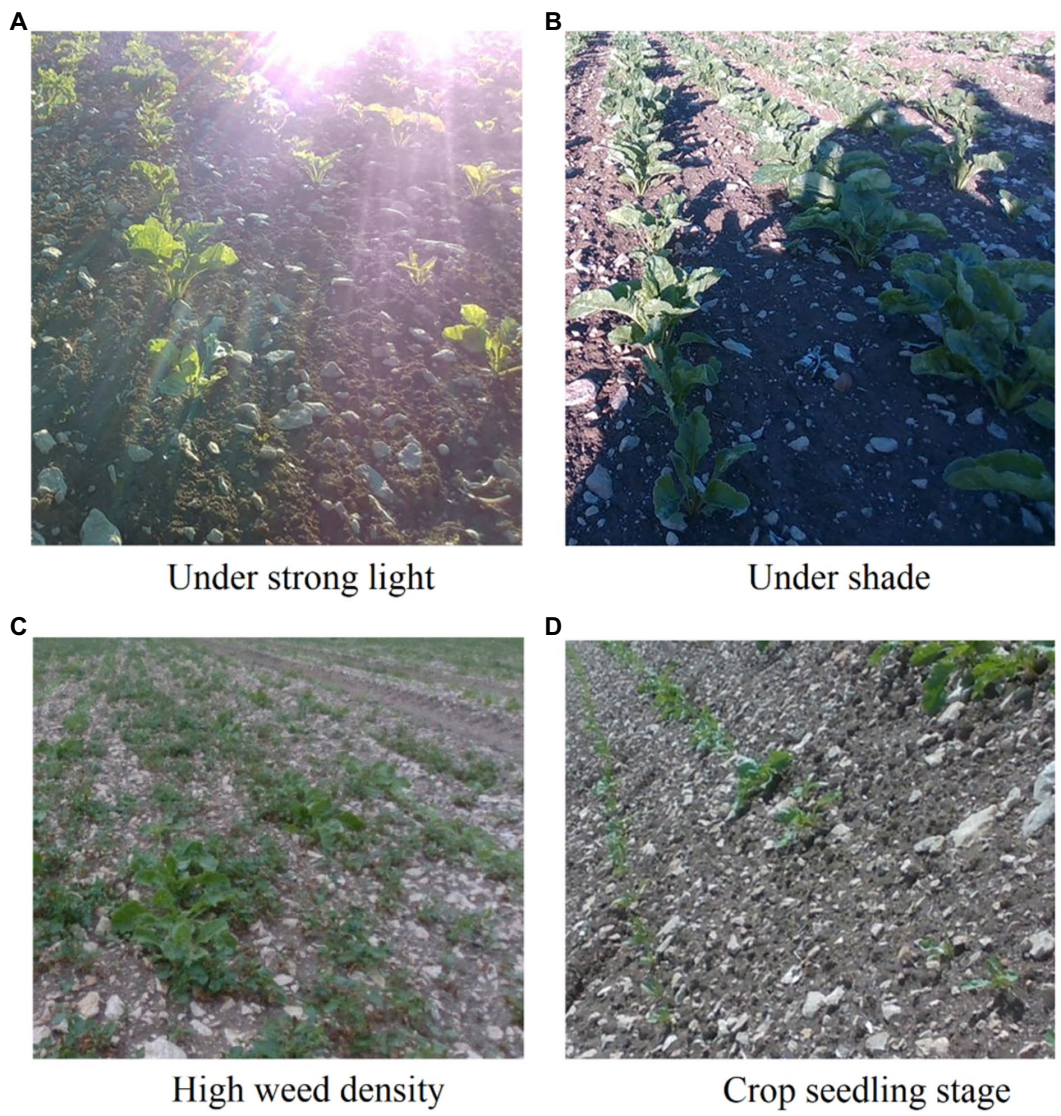
Crop row image in a complex environment. **(A)** An example of a crop row image in a strong light environment. **(B)** Example crop row image with large areas of shadow. **(C)** Example crop row image with lots of weeds. **(D)** Example crop row image at seedling stage.

We used the publicly available The Crop Row Detection Lincoln Dataset (CRDLD; de Silva et al., 2021), which is a dataset generated from RGB images obtained using an Intel RealSense D435i camera under different weather conditions and different times of the day in a beet field. As shown in Figure 5, the dataset collects basic images for different values of sunlight exposure, shade, weed density, and crop growth stages. These complex scenarios put forward high requirements for the robustness of the model.

**Figure 6:**
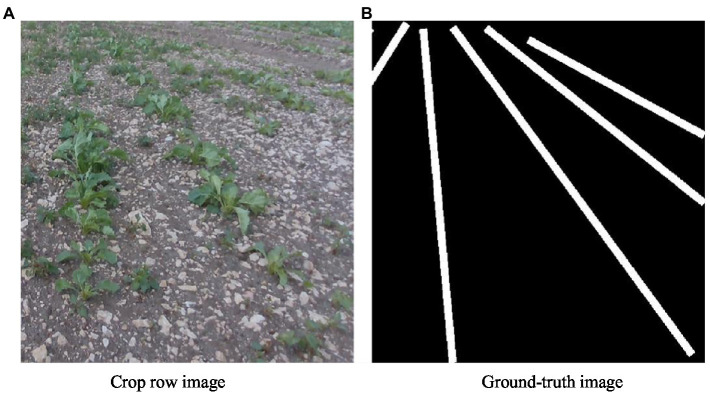
Sample crop row images and their corresponding ground-truth images.

After the acquisition is completed, the images in the dataset are expanded by cropping the images in four directions and are then labeled. The dataset contains 1,000 training images and 100 testing images. The crop row images in the dataset and their corresponding ground-truth images after row segmentation are shown in Figure 6.

### Crop Row Segmentation

For the experiments, we implemented commonly employed semantic segmentation networks under the PyTorch deep learning framework, including FCN8S, SegNet, ERFNet, UNet, ENet, and an improved model based on the ENet network. The FCNS model replaces the fully connected layer behind the traditional convolutional network with a convolutional layer, while simultaneously adopting the method of cross-channel addition to enhance the transferability of information. FCN8S performs downsamples for a maximum of eight times in the network. The network structure of SegNet is similar to that of FCN. Nevertheless, the first 13 layers of the VGG-16 structure are used in the encoding part, and the upsampling in the decoding part uses spatial index information. UNet is a U-shaped symmetric structure, and skip connections dimensionally splice the feature maps. ERFNet is an improvement to the ENet network, with core operations of residual connections and factorized convolutions.

The network training process involves training and testing the model using pre-divided data, including 1,000 training images and 100 testing images. The size of the input image is 512×512. The following hyperparameters were used for network training: an initial learning rate of 0.0001, and a learning rate decay coefficient of 0.1. The network was trained for 85 epochs, with eight samples per iteration. The model was trained using standard gradient descent, and the optimization method was the Adam adaptive learning rate optimizer.

In order to improve the performance of the model and avoid overfitting of the model during the training process, this paper uses random horizontal flip, random vertical flip, and random 90° rotation for data augmentation during model training. The image changes with a probability of 0.5 each time the image is read for each iteration of training.

The training process used was the BCEDiceLoss function, which is a combination of binary cross-entropy loss and dice loss. The calculation formula of the loss function is as follows:


(4) BCEDiceLoss=BCELoss+DiceLoss

where the calculation formula of the binary cross entropy loss is as follows:


(5) $$\begin{array}{l} \mathrm{BCELoss}\left(y,\hat{y}\right)\\ =-\frac{1}{m}\overset{m}{\underset{i=1}{\sum }}\left[y_{i}\log S\left(\hat{y_{i}}\right)+\left(1-y_{i}\right)\log\left(1-S\left(\hat{y_{i}}\right)\right)\right] \end{array}$$

(6) $$S\left(x\right)=\frac{1}{1+e^{-x}}$$

where *y* represents the pixel value of the ground-truth image, $\hat{y}$ represents the pixel value of the predicted image, and *m* represents the total number of pixels in the image.

The calculation formula of dice loss is given as follows:


(7) $$\mathrm{DiceLoss}\left(Y,\hat{Y}\right)=1-\frac{2\left|Y\cap \hat{Y}\right|+\mathrm{smooth}}{\left|Y\right|+\left|\hat{Y}\right|+\mathrm{smooth}}$$

where *Y* represents the true value image, $\hat{Y}$ represents the predicted image, and *smooth* is a parameter added to prevent the denominator from being 0. If this parameter is too large, the calculation result of DiceLoss will be slightly higher. Experiments showed that setting *smooth* to 0.1 can reduce the influence of this parameter on the final loss value.

**Figure 7:**
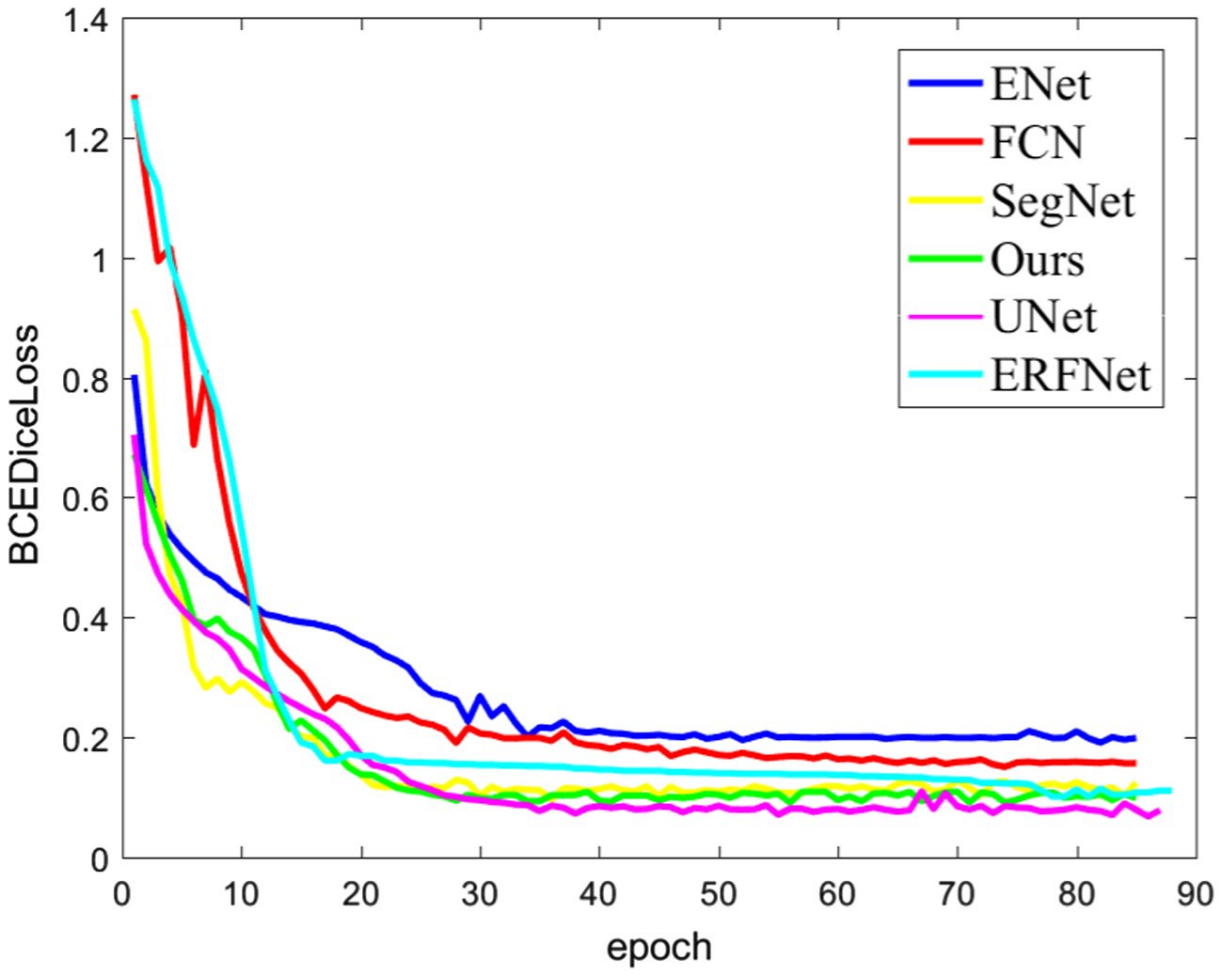
Loss function curve.

The relationship between the loss function value of each network model and the epoch during the experimental training process is shown in Figure 7. The figure shows that with an increase in the training times, the fitting effect of the model improves continuously, and the model is gradually optimized. A comparison of the loss function value curve of each network model shows that the proposed model has a faster convergence speed and a smaller loss value. That is, the model has a higher segmentation accuracy.

The appropriate evaluation metrics that accurately analyze each network model’s performance must be selected. The proposed segmentation network model is dedicated to improving the accuracy of extracting visual navigation lines for agricultural flying robots while ensuring good real-time performance. We chose the intersection over union (IoU) as the criterion to determine whether the crop row is correctly detected. The IoU is a standard measure in semantic segmentation tasks, and it is calculated as follows:


(8) $$IoU=\frac{H\cap T}{H\cup T}\;\times \;100\%$$

where *H* represents the predicted value range, and *T* represents the actual range. The higher the IoU value, the higher the coincidence between the predicted range and the actual range, that is, the higher the segmentation accuracy. In this study, if the IoU was greater than or equal to 0.6, it was determined that the crop row was correctly detected, and incorrectly detected otherwise. The speed at which the model ran was measured in terms of image frames per second. Considering the feasibility of deploying the model on limited embedded devices, model parameters must be added to measure the computational complexity of the model.

**Figure 8:**
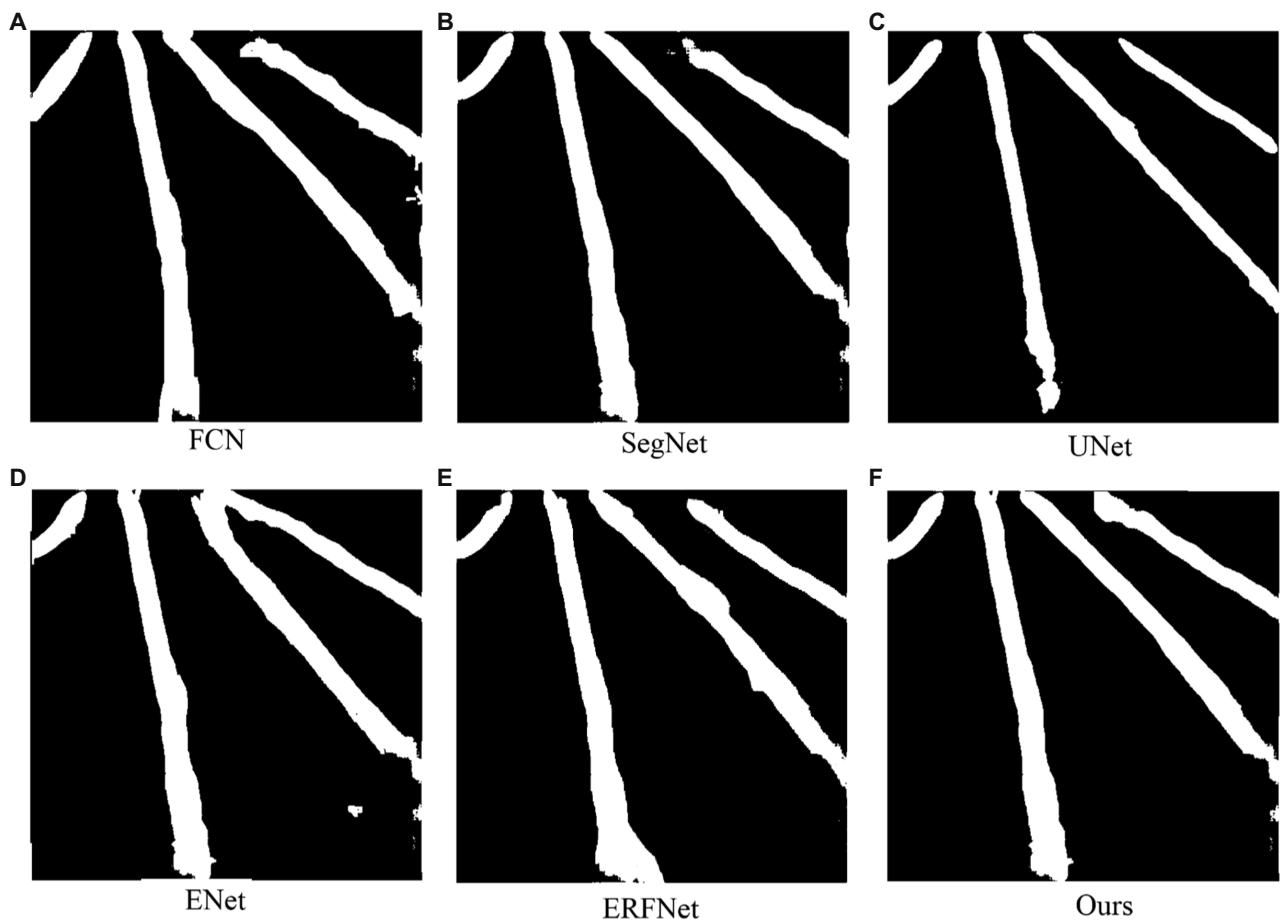
Example segmentation results for each trained model. **(A)** Example of FCN model segmentation results. **(B)** SegNet model segmentation result example. **(C)** UNet model segmentation result example. **(D)** ECN model segmentation result example. **(E)** ERFNet model segmentation result example. **(F)** This paper proposes an improved model segmentation result example.

**Figure 9:**
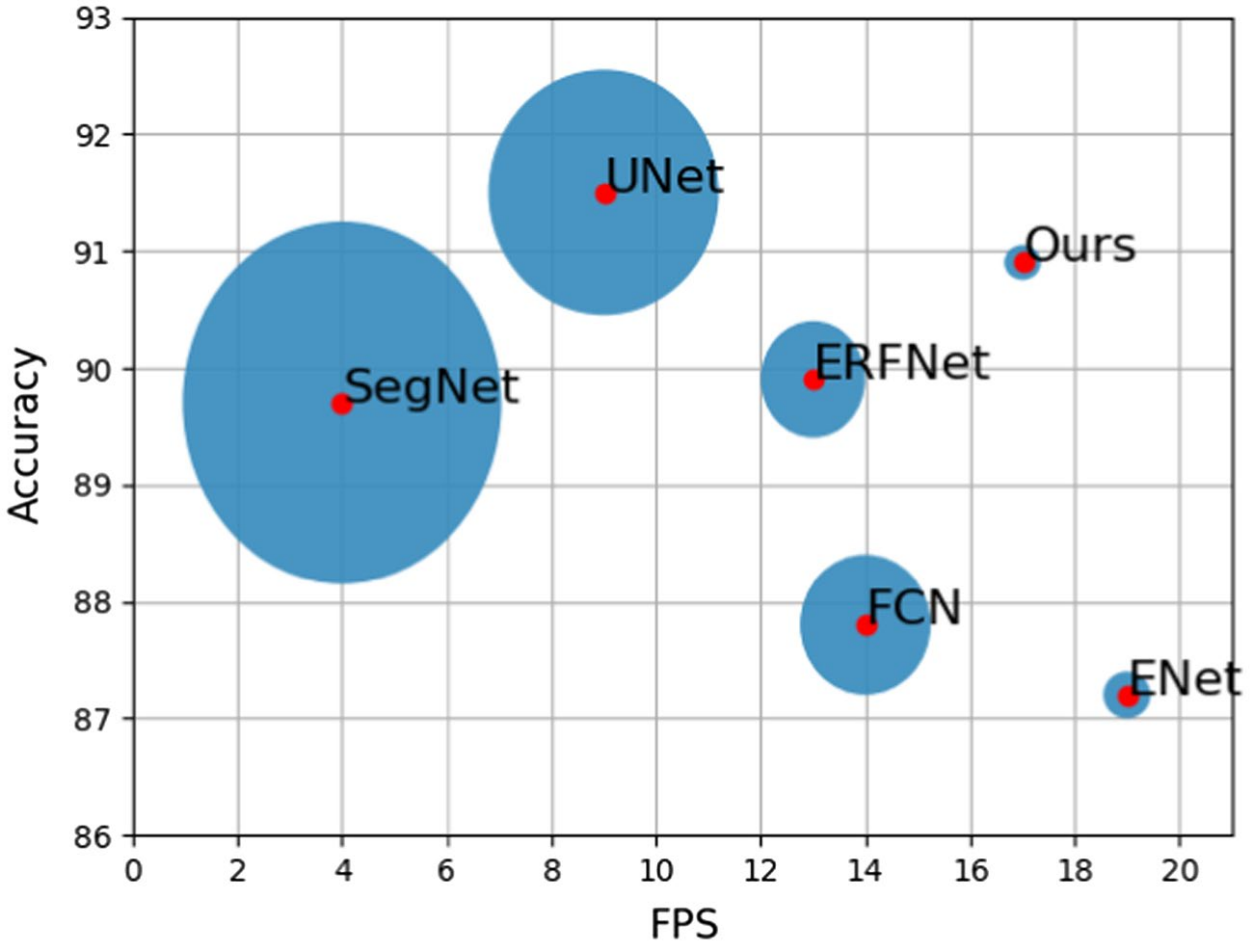
Performance comparison of various algorithms.

After the semantic segmentation model is trained, segmentation results can be obtained by inputting the test images. The visualization of each model training result is shown in Figures 8A–F. Table 1; Figure 9 show the performances of the different models. In the figure, the solid circles represent each network model, the abscissa is running speed of the model, denoted by FPS, the ordinate is the model segmentation accuracy, and the size of the circle depicts the parameter quantity of the model, that is, the computational complexity of the model.

**Table 1:** Performance comparison of different models.

Model	Accuracy/%	FPS	Parameter/MB
FCN8S	87.8	15	5.2
SegNet	89.7	4	29.4
UNet	91.5	9	18.4
ENet	87.2	19	0.34
ERFNet	89.9	13	2.1
Ours	90.9	17	0.27

The experimental results show that the segmentation accuracy of the designed network model is improved compared with FCN, ERFNet, SegNet, and classic ENet. Although it still does not match the accuracy of UNet, it only differs by 0.6%. Operation speed is reduced because of the addition of residual branches to the classic ENet structure. However, it is still faster than those of FCN, SegNet, ERFNet, and UNet. In terms of computational complexity, the designed network model is much smaller than UNet and SegNet. The model has 0.05% the parameters of the FCN model, and it only increases the parameter amount by 0.07 M compared to classic ENet. Therefore, the model is still a lightweight network that rapidly performs semantic segmentation. From the performance comparison chart shown in Figure 9, it can be seen that the closer the model position is to the upper right, the stronger the overall performance. Therefore, the performance of the proposed network is better than that of the other five semantic segmentation networks, improving the segmentation accuracy without reducing the real-time performance and achieving the expected effect.

### Navigation Line Recognition

**Figure 10:**
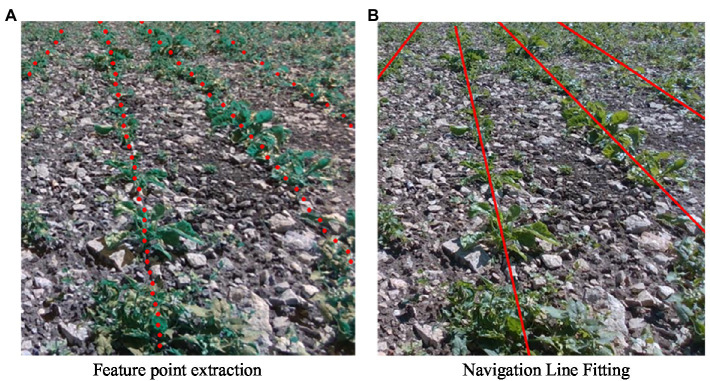
Guideline fitting effect. **(A)** An example of the result of feature point extraction, where the red dots represent the extracted feature points. **(B)** An example of the results of the guideline fit. The red line in the figure is an extremely fitted visual navigation line.

We extract and fit the feature points of the image segmented by the network to obtain a visual navigation line, as shown in Figure 10, where the red dots in Figure 10A represent the extracted feature points, and the red lines in Figure 10B are the fitted crop row centerlines.

To quantitatively analyze the accuracy of guideline detection, we drew guidelines manually from 100 ground-truth images in the test dataset used as the reference standard. We used the method proposed by Jiang et al. (2015) for the detection results of the navigation line of the crop row, which compares the predicted results with the artificially drawn true values. The angle α is calculated by comparing the predicted result with the manually drawn true value. When α is not greater than 7°, the algorithm’s detection result is considered to be correct; if it is greater than 7° or is not detected, the result is considered incorrect.

**Figure 11:**
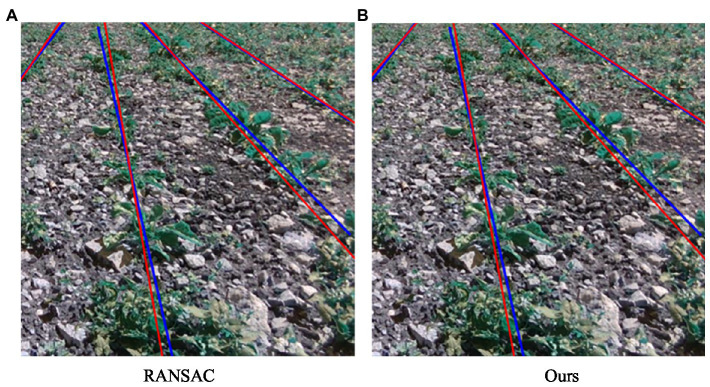
Navigation line fitting results. **(A)** The red straight line represents the navigation line fitted by the RANSAC algorithm. **(B)** The red straight line represents the crop row navigation line fitted by our algorithm.

The RANSAC algorithm and the guideline fitting algorithm proposed here were used to detect the guidelines. Table 2 provides a performance comparison of the two algorithms, and the detection results are shown in Figure 11. The detection accuracy and the red line in the figure represent the crop line, and the blue line is the manually calibrated navigation line, that is, the true value of the navigation line.

**Table 2:** Performance comparison of the fitting algorithms.

Algorithm	Average time/ms	Accuracy/%
RANSAC	98	90.8
Ours	55	91.2

A comparison of the results of the two fitting algorithms is shown in Table 2; we can see that the accuracy of the improved RANSAC algorithm proposed here is lower than that of the RANSAC algorithm, but the overall time consumption is considerably improved, and the total time is reduced by 44 ms. Overall, the straight-line fitting algorithm proposed here is more optimal, which ensures the algorithm’s real-time performance without losing accuracy.

## Conclusion

Considering the inaccuracy and poor robustness of traditional visual navigation line extraction algorithms, we proposed a visual navigation method based on an improved semantic segmentation network, namely ENet. Using the trained semantic segmentation network training dataset, we extracted feature points from the segmentation results and classified them. Finally, we used a random sampling algorithm to fit the centerline of the crop row as the visual navigation line. The experimental results showed that the algorithm has good adaptability to farmland images in various situations. The accuracy of crop row detection was 90.9%, the FPS of the model was 17, and the number of parameters was 0.27 M. Therefore, it can be deployed in embedded devices, which can meet the actual requirements and allow follow-up agricultural UAVs to realize real-time and precise flights along the ridges in farmland operations.

The study conducted an experimental test on an existing dataset, and there may be various scenarios in an actual farmland environment. Therefore, in future work, more pictures of different growth states and environments will be collected on the spot to expand the dataset, and further research will be conducted. This will improve the practicability of the model and promote the application of semantic segmentation in intelligent visual navigation.

## Data Availability Statement

Publicly available datasets were analyzed in this study. This data can be found at: https://github.com/JunfengGaolab/CropRowDetection.

## Author Contributions

MC: conceptualization, methodology, investigation, formal analysis, and writing—original draft. FT: investigation, software, and writing—original draft. PJ: validation and writing—review and editing. FM: conceptualization, funding acquisition, supervision, and writing—review and editing. All authors contributed to the article and approved the submitted version.

## Funding

This work was supported by the National Natural Science Foundation of China, 61903207, the Key Research and Development Plan of Shandong Province, 2019JZZY010731, and Youth Innovation Science and Technology Support Plan of Colleges in Shandong Province, 2021KJ025.

## Conflict of Interest

The authors declare that the research was conducted in the absence of any commercial or financial relationships that could be construed as a potential conflict of interest.

## Publisher’s Note

All claims expressed in this article are solely those of the authors and do not necessarily represent those of their affiliated organizations, or those of the publisher, the editors and the reviewers. Any product that may be evaluated in this article, or claim that may be made by its manufacturer, is not guaranteed or endorsed by the publisher.
