# Corrigendum: Improved real-time semantic segmentation network model for crop vision navigation line detection

**DOI:** 10.3389/fpls.2023.1140560

**Published:** 2023-02-09

**Authors:** Maoyong Cao, Fangfang Tang, Peng Ji, Fengying Ma

**Affiliations:** School of Information and Automation Engineering, Qilu University of Technology (Shandong Academy of Sciences), Jinan, China

**Keywords:** precision agriculture application, visual navigation, semantic segmentation, crop rows detection, navigation path recognition

In the published article, the reference for "Brostow et al., 2008" was incorrectly written as “Brostow, G. J., Shotton, J., Fauqueur, J., and Cipolla, R. (2008). “Segmentation and recognition using structure from motion point clouds.” in European Conference on Computer Vision. Berlin, Heidelberg, (Springer), 44–57”. It should be "de Silva, R., Cielniak, G., and Gao, J. (2021). Towards agricultural autonomy: crop row detection under varying field conditions using deep learning. arXiv [Preprint] arXiv:2109.08247".s

In the published article, the reference for “de Silva et al., 2021” was incorrectly written as “de Silva, R., Cielniak, G., and Gao, J. (2021). Towards agricultural autonomy: crop row detection under varying field conditions using deep learning. arXiv [Preprint] arXiv:2109.08247”. It should be “Brostow, G. J., Shotton, J., Fauqueur, J., and Cipolla, R. (2008). “Segmentation and recognition using structure from motion point clouds.” in European Conference on Computer Vision. Berlin, Heidelberg, (Springer), 44–57”.

The authors apologize for this error and state that this does not change the scientific conclusions of the article in any way. The original article has been updated.

